# Self-management experiences of youth following the unexpected loss of a family member to HIV

**DOI:** 10.4102/hsag.v27i0.1751

**Published:** 2022-04-28

**Authors:** Siphesihle D. Hlophe, Karien Jooste

**Affiliations:** 1 Department of Nursing Science, Faculty of Health and Wellness Sciences, Cape Peninsula University of Technology, Cape Town, South Africa

**Keywords:** bereavement, comprehensive primary healthcare, death, family, grief, HIV, outpatient, youth

## Abstract

**Background:**

Death of a close family member is one of the most traumatic events in a person’s life. The way, in which this loss unfolds, varies from person to person and depends on how close you were with the deceased. It was unclear how youths experienced it to manage themselves during different stages of the bereavement process, after losing a near-family member to human immunodeficiency virus (HIV).

**Aim:**

The aim of this study was to understand the self-management of youth following the unexpected loss of a family member to HIV.

**Setting:**

Khayelitsha, Western Cape province, South Africa.

**Methods:**

A descriptive phenomenological design was followed, with an accessible population of youth who lost a family member to HIV. Individual semi-structured interviews were conducted with 11 purposively selected participants after obtaining written informed consent. The sessions, held with an interview schedule, did not take longer than 45 min to conduct until data saturation was reached. A digital recorder was used and field notes held. Open coding was followed after transcribing interviews.

**Results:**

Individuals had different experiences during different stages of the bereavement process, not necessarily similar or following the same sequence. Individuals had to manage their guilt of being unable to do more before the family member passed away, struggling to realise that they have a future purpose, and hardship having fond memories.

**Conclusion:**

Youth find it difficult to view death as a natural loss of life and to manage themselves after the loss of their loved one to HIV.

**Contribution:**

The context-based information in this study confirms the importance of youth and self-coping and self-continuation to plan, organise and direct their future after the loss of a family member.

## Introduction and background

A family is often a source of emotional support, love, security and protection, and may provide a unique sense of belonging and values that cannot be found in other relationships (Revilla 2019). Youth going through the unexpected death of a family member should be assisted with handling grief and establishing a positive mindset about realising their future.

According to Matthews (2017), when it comes to grieving the death of a loved one, there are no linear patterns, no ‘normal’ reactions and no formulas to follow. The reality of death affects families in a myriad of emotional or physical ways while also shifting family systems and impacting spirituality. Lekalakala-Mokgele (2018:151) explains that each person’s grief is unique and does not follow any pattern or path. Youth could have different ways of showing grief, such as crying, fear and anger, which are common and universal; however, the cultural environment should also be considered when complicated grief is experienced. Most bereaved people can overcome their grief; however, in some cases, grief becomes prolonged or complicated (Wilson, Underwood & Errasti-Ibarrondo 2021:1).

Individuals who lose a family member go through various stages of grief, which they may not always fully understand. In 2017, Stroebe, Schut and Boerner stated that it can frequently be interpreted as a prescription of progress that bereaved persons must implement to get to terms with loss. However, authors mention that stages are often time-related, and each stage is necessary to reach the following stage (Selman et al. 2021:1267). Different people experience grief in different ways, not necessarily cycling through the stages to overcome their grief, and stages of grief can overlap (Fletcher 2020). In this process of change, grieving people such as youth almost always need the support of others. The common thread for all grieving people is change (De Andrade Alvarenga et al. 2021:434). Not only do they change inwardly but their daily routine changes leading to a measure of adjustment, which can be hard to deal with (Wang & Wang 2021:79).

Someone who unexpectedly loses a family member can move on a continuum range of emotions such as from denial to acceptance (Özel & Özkan 2020:353). In the process of taking ownership of playing a new role in assisting others (who were previously supported by the deceased), they, sometimes, give up their own dreams (Wang & Wang 2021:80). Sad experiences could block out memories of good times they had with the deceased (Owen 2021:1). When you are grieving, it is more critical than ever *to take care of yourself*. The consequences of a traumatic incident can rapidly exhaust your energy and emotional reserves. Looking after one’s physical and emotional needs will assist one to get through a troublesome time (Smith, Robinson & Segal 2020) and manage oneself.

Self-management, that *is the act of practising intentional self-care,* could thus help youth to move through different stages of grief, to get stronger and fill themselves with the hope for what lies ahead (Wolf 2019).

Lenzen et al. (2017:1) define self-management ‘as the degree to which persons have the ability to control their own lives and to cope effectively with adjustment, such as grief’. When a family member passes away successfully, self-management could be demonstrated by dealing with the stages of grief towards improving one’s health status (Crowley et al. 2019). The management of oneself could decrease distress associated with a life event such as losing a family member. Empirical evidence confirms that health outcomes are enhanced in individuals such as youth, who engage in self-management, using condition-specific knowledge, beliefs and self-regulation skills (Crowley et al. 2019). Youth are defined as persons between the ages of 18 and 25 years in the state or quality of being young, energetic and immature (The United Nations Educational, Scientific and Cultural Organization [UNESCO] 2017).

Going through stressful times and stages is part of life; however, youth may feel unfocused, overwhelmed and helpless (Griffin 2019). Youth who have lost their parents may also increase their alcohol use, and their relationship with siblings may be positively or negatively affected (Glatt 2018:115).

An active approach (including cognitive and behavioural strategies) develops self-management skills via self-reflection, solving problems and active goal setting (Dineen-Griffin et al. 2019:11). Individuals must not discontinue practising self-management strategies, regardless of their emotional pain, as it is an ongoing healing process that is crucial for the development of the ability to separate the ‘self’ from pain and to develop self-efficacy (Devan et al. 2018:393).

Practising intentional self-management can help family members to feel stronger, restore their sense of peace and fill one with hope for what lies ahead (Wolf 2019). In this study, it referred to the planning, organising, directing and control of the youth dealing with the loss of their family members.

It is essential to understand the experiences causing pain and grief to individuals in order to optimise the design and delivery of self-management interventions (Devan et al. 2018:382). Youth could experience emotions such as fear and depression after the loss of a family member with, for example, human immunodeficiency virus (HIV). In some families, a young person must then act and behave as a parent when the actual parent passes away (Glatt 2018:107). People who are experiencing grief are very likely to be depressed and consequently less likely to engage in active self-management strategies (Devan et al. 2018:394). It was observed that some youth visiting a public comprehensive primary healthcare clinic in Cape Town experienced panic attacks, anxiety and a loss of direction as a result of losing a family member. It was, therefore, unclear what the lived experiences were of youth, managing themselves after the loss of a family member to HIV.

## Purpose

The purpose of this study was to explore the lived experiences of youth on self-management following the loss of a family member with HIV.

### Design

Descriptive phenomenology was employed, which allowed the researcher to gain an in-depth understanding about the phenomenon of losing a family member to HIV.

The accessible population was youth who visited a comprehensive primary healthcare clinic in Cape Town of the Western Cape in February 2020. Purposive sampling was followed, and participants had to meet the inclusion criteria of losing a close family member to HIV, who lived in the same house hold, for a period of last 6 months. Eleven (*n* = 11) semi-structured individual interviews were conducted with the enquiry method, using an interview guide, that lasted around 45 min. The use of probing questions led to data saturation and yielded insights into the lived experience of the youth (Ellis 2019:48).

### Data gathering

Participants were recruited at a comprehensive primary healthcare clinic between January and February 2020 according to an agreement made with the professional nurse in charge. A poster placed at the entrance of the comprehensive primary healthcare clinic invited participants to partake in the study. Staff also referred patients (youth), after completing the health consultations, to the waiting area outside a private room where the interviews were conducted. Participants were interviewed at a clinic room on site, where there were no disturbances. The researcher asked participants that he could keep field notes and use a digital recorder during the interviews.

### Data analyses

The recordings were transcribed. Some of the interviews had to be translated. Those in the local language (isiXhosa) were translated into English by the researcher and back translated by an editor who also spoke both languages to ensure dependability of the data interpretation. Data transcription began by establishing the unit of analysis to be studied, addressing which information should be included in a transcription. Interview data and field notes were coded simultaneously. An independent coder was used who held a consensus meeting with the researcher on the themes and categories that emerged from the data.

### Trustworthiness

The trustworthiness of the study was ensured in different ways. Credibility was ensured through triangulation as it involved the use of different data collection methods of interviews and field notes, in order to ensure consistency of the findings. Dependability was ensured by using an independent coder who analysed the data and results of the study and had a consensus meeting with the researcher. In order to ensure confirmability, the data reflected the voices of the participants, techniques of inquiry audit, reflexivity (fairness in inclusion criteria) and triangulation of data (interviews and field notes). Transferability in the study was established by providing a thick description of the methodology and findings as evidence that the research study’s findings could be applicable to other contexts, situations, times and populations. The applicability of the findings was limited to female participants.

### Ethical considerations

The scientific methodology and ethics of the research project entitled: Youth need beseech self-management to face the difficult stages after the unexpected loss of a family member to HIV, was approved for the period: January 2020 – December 2021 by the Ethics Committee of the Cape Peninsula University of Technology.

Permission was obtained from the Research and Ethics Committee of the Faculty of Health and Wellness (Ethics clearance number: CPUT/HW-REC 2019/H2) and the Department of Health in the Western Cape Province (Ethics clearance number: WC_201911_032). Participants received an information sheet in their language of choice (English, Xhosa). The study was verbally explained and their questions answered. Participants participated voluntarily and could withdraw from the study at any time, without any consequences. Confidentiality was maintained as names of participants in the interviews remained anonymous on the transcripts. Harm to the participant was minimum (some showed some emotions, crying); however an advanced psychiatric nurse practitioner was arranged to be available at the clinic, in the case that a participant needed emotional support, and had to be referred. Data were stored online with password protected files on the main researchers’ computer. It is planned that all data will be destroyed five years after the publication of the report of the study (South Africa 2003).

### Results

Participants (*n* = 11) were limited to black women aged 18–25 years, including the participant in the pilot interview. They had lost a near family member to HIV. The relationships with those whom they lost were their mothers (*n* = 2), grandmothers (5), aunt (*n* = 2), sister (*n* = 1) and a brother (*n* = 1). Six participants had their own children, previously taken care by the deceased, while five participants had to take care of their children left behind by the deceased. Six of the participants were attending school or a college, and five were unschooled and in the working class.

Four themes came to the fore, and the results will *focus on* the specific theme self-management experiences of youth following the unexpected loss of a family member to HIV.

The categories that emerged under the theme of stages after the unexpected loss of the family member were related to:

1. denial and grief,
2. guilt at being unable to do more before the family member passed away,
3. a struggle of those left behind to realise that there is a future,
4. hardship of losing someone dear about whom one has fond memories and
5. yearning for the deceased who had a positive effect on their lives.

#### Denial and grief

The common emotions related to grief are shock, fear, confusion and loneliness (Glatt 2018:109; Lekalakala-Mokgele 2018:151). It was mentioned that in some cases, family members had passed away without disclosing their HIV status, which led to shock among some participants:

‘I was failing to accept the situation … because my sister didn’t eat her treatment well and she was hide that she is positive. I also find out that she died because of HIV when she passed away.’ (Angry face) (P9, female, 21 years old, college student)

Some of the participants experienced pain and hurt at the loss of their family member. It came unexpectedly and resulted in a failure to accept the situation. A participant said:

‘It was very painful. Because it’s something that comes unexpectedly.’ (Tearful) (P6, female, 23 years old, health promoter)

Death is certain to happen, but this did not make it easier for those left behind. A participant spoke of her denial of the death:

‘I was not accepting it at all. I was not accepting it and … yho….’ (P8, female, 22 years old, municipality worker)

The pain of losing someone close can be experienced as unbearable. Unlike other pain, death can cause a heartache that is sometimes carried to the grave (Drake 2019).

#### Guilt at being unable to do more before the family member passed away

Guilt manifests in a negative evaluation of one’s own behaviour and a sense of remorse at not having done more, a failure to adapt and an inability to cope with stress after the loss of a loved one (Slepian, Kirby & Kalokerinos 2020:323). In one of the participants, the failure of the deceased, a sister, to reveal her HIV status led to a participant feeling guilty that she had not done more to support her sister and possibly prevent the loss:

‘Because if she would tell us, there was nothing wrong to be HIV positive. We understand, we could have managed to tell her to go to the clinic all the time, but she was not going to the clinic.’ (P6, female, 23 years old, health promoter)

Living with a sense of guilt is difficult and can make it hard to get through each day, and could need support by a therapist. However, some participants felt angry and blamed the deceased for their sudden death. These feelings were coupled with a sense of guilt in the following participant who had lost her brother. It bothered her that various ways of appropriate support could have been provided to the brother had the family known:

‘You know that I always blame my brother, neh, for not telling me. Maybe I was going to do something better if he told me his status. But he didn’t. Why? I don’t know. Maybe I’ll find information and places where he can get help. Maybe he didn’t die – maybe he shouldn’t die by that time.’ (P10, female, 25 years old, college student)

A participant mentioned that she had guilt feelings about her mother, such as not appreciating her enough, as when peers spoke about their mothers, she could not participate in the conversation:

‘I was having stress because other children at school were talking about their mothers. Maybe they say my mother buy me this and that, while I was feeling guilty but ekuhambeni kwexesha nda right (as time went by, I became alright).’ (Sad face) (P8, female, 22 years old, municipality worker)

#### Struggle of those left behind to realise that there is a future

Adolescents who are still at school after losing a family member often experience a sharp decline in their academic performance, emotional health and social functioning. The struggle to move forward affects a number of daily activities, including sleep, interpersonal relationships and attending to daily activities such as school activities, all of which can lead to drug and alcohol abuse (Adams 2018:4). A participant mentioned that she had lost a sense of the future because of her fearfulness following the death of her mother:

‘… [*S*]o I felt like also my dreams have come to an end. I will not be able to pursue my dreams since I have to take over as a breadwinner at home. Those were my fears.’ (Points to her chest) (P1, female, 24 years old, pharmasist assistant)

The loss of a loved one who played the role of breadwinner in the family can result in fear about future responsibilities that are now passed to the older children. The eldest child may fear being unable to cope with financial duress.

In the case of the following participant, the loss of her brother led to a loss of a sense of purpose in life and thoughts that she had lost everything. Because of the sense of devastation and loss of purpose, she resorted to self-destructive behaviour:

‘So, after he passed away I lost everything. By trying to forget him I just wanted to drink alcohol and smoke cigarettes.’ (Looks down, avoids eye contact) (P10, female, 25 years old, college student)

Losing a brother that loved one can deeply feel like the loss of everything:

‘When I’m talking about losing everything I’m talking about love. I didn’t find love the way he (the brother) did, the way he used to love me.’ (Becomes emotional) (P10, female, 25 years old, college student)

#### Hardship of losing someone dear about whom one has fond memories

The hardship of parental loss is reported to have higher chances of causing mental health problems to the bereaved individuals than the loss of other family members (Rosenbaum-Feldbrügge 2019:1828). Participants mentioned that losing someone they loved and had shared memories with was hard. They spoke of thinking a lot about the person’s good qualities and the good things the person used to do for them:

‘You know, it’s not nice when a person you depend on passes away.’ (P2, female, 25 years, college student)

Another participant mentioned that it was hard for her as her sister left two children behind:

‘It’s not easy to lose somebody that you love, it’s not very easy. So, the pain is that she had only two children.’ (Rubs hands on thighs) (P6, female, 23 years old, health promoter)

One of the participants stated that the brother used to do all the chores at home, including her homework. She said:

‘He used to do everything for me, my homework, wash my clothes and preparing school things for me.’ (P10, female, 25 years old, college student)

Individuals seem to be trying to fill a gap by engaging in negative behaviour instead of looking forward and viewing that their life still has a purpose. A participant mentioned that her brother had been her anchor in life, and she was having difficulty in forgetting him:

‘Just to try and forget about him because he was there, he was everything to me.’ (P10, female, 25 years old, college student)

Family members may sense an emptiness where that person once was, as they provided emotional support and played a significant role in the daily routines of the family members (Rosalia 2016).

#### Yearned for the deceased who had a positive effect on their lives

Some participants mentioned the good and positive things that the lost family member had inculcated in them. These family members had made them feel comfortable, positive and self-confident. The loss resulted in a devastating change in self-perception. One individual said that she sometimes did not want to go home because her brother was not there:

‘Sometimes I didn’t wanna go home just because I know, he used to cook for me. And, we used to sing for each other and pray together. So, there was no one who did that with me after he passed away. I ended up to live with friends at the street.’ (Voice shakes) (P10, female, 25 years old, college student)

The loss of her sister made a participant longing for her no other option but to look for a job in order to take care of herself. Her father seemed unable to provide for her either financially or emotionally. She said:

‘I miss her a lot (emotional face and tears in the eyes). I always think about her now. When my life is going down and up, I end up find myself to go and look for job, but there’s no one to comfort me anymore besides my dad.’ (Emotional face) (P4, female, 24 years old, a learner in Grade 11)

She also mentioned that she missed her sister who used to make her feel comfortable and with whom she shared life challenges and experiences. She felt that she had lost a part of herself with the loss of her sister:

‘She was a grocer. She was the one who carries me when I’m in a bad mood, and I talk. She was the one who makes me comfortable when I want to talk. And she was the one who ask me about school, my things in life. I was feeling comfortable but now I don’t have anyone to talk to. I am scared to talk to my father how I feel. I lost very much, and I still miss her until now.’ (Sits all the way back on the seat.) (P4, female, 24 years old, a learner in Grade 11)

A participant mentioned that her sister had been an extrovert person:

‘So, I was very sad because she was a very talkative person.’ (P6, female, 23 years old, health promoter)

A person, who was HIV positive herself missed her grandmother who used to make her feel positive and confident regardless of her condition:

‘For me it was really hard. … Hmm … first of all, I am also infected with HIV/AIDS and then my grandmother was the one who gave me support. Eh! She was the one who made me feel positive. The one who gave me like the spirit to have confidence in myself. Not to compare myself with other children.’ (P5, female, 19 years old, a learner in matric)

In the case of the above participant, the potentially negative experience of being found to be HIV positive was possibly ameliorated by the positive impact of the now deceased grandmother on her life.

The participant went on to say that words of encouragement from her grandmother had motivated her to the point that she still felt that she was no different to others, despite her HIV status. In other words, her grandmother’s positive influence remained with her, to some extent:

‘Like (takes a deep breath) she would convince me to, show me ways and come up with examples, like I’m like other children. I’m not different. I can do whatever I want to. Since I have this disease, I am not different to others. Like she tried by all means, showing me that I’m not differ to others.’ (P5, female, 19 years old, a learner in matric)

Her grandmother had provided her motivation, and there was now a need to keep that motivation and make it self-motivation. Self-motivation is the drive to work towards one’s objectives, to put exertion into self-development and to realise individual fulfilment (Ackerman 2020). These refer to cognitive behavioural therapy.

She mentioned that her grandmother convinced her to be in a relationship and gave her advice with family planning methods:

‘Firstly, since my grandmother was positive, and I was also positive. Like I was comparing myself. I didn’t want to go out, but my grandmother would convince me to. I was scared dating, but she is the one who gave me ideas, if I want to date I must go and prevent, I must not be ashamed of what I have. And, even at school I must be a kid. Ja, for now …’ (no eye contact, looking down and biting nails) (P5, female, 19 years old, a learner in matric)

It was clear that family connections are significant for one’s well-being over the course of life.

## Discussion

The loss of an individual could be understood as a natural part of life; however, those left behind are still overcome by shock and confusion, leading to prolonged periods of sadness or depression. Testoni et al. (2020:1) concur that *grief* is an inescapable part of life because death is inevitable. They state that for human beings, grief is a natural reaction to loss, and can be expressed in different forms depending on the individual. However, all these stages are part of the grieving process, which is important to go through in order to overcome the negative feelings and begin to embrace the good times one had with a loved one (Nordal 2020).

The loss of a family member seemed to create an environment of many forced changes that requires adapted behaviours to stabilise circumstances. Some individuals in the families were greatly affected by the loss of a family member, as they are more senior family members, needed to take on an authoritarian position to support siblings. These role models, sometimes, had to take on responsibilities at an early age, while they themselves need support to act strong and follow in the footsteps of the deceased, for example, parents. According to Griefic (2020), there are several signs and symptoms of early acute grief, with loss of focus and concentration of the most common indicator. The findings indicated that youth experienced a variety of emotions related to death, depending on their relationship with the deceased, and the manner of the death (HIV or AIDS). The pattern of grief was unique to each participant and the components of grief were identified, not necessarily following one pattern such as failing to accept the situation they faced or experience the loss of affection previously.

After the death of a loved one, some participants were left behind, facing the challenge of dealing with a sense of guilt and self-blame. ‘Guilt’ is a feeling that one could have done something to prevent the death of the loved one (Li, Tendeiro & Stroebe 2019:457). Those with a sense of guilt struggled to connect with their family and experience an inability to focus on their work or school activities. According to Slepian et al. (2020:323), guilt could be associated with shame that is a negative evaluation of the self and feeling helpless or small.

Participants struggled to realise that there is future and older children who had to make major changes in their life, such as paying bills, taking care of younger siblings and getting a job that paid better than their existing job. Children with a far greater sense of responsibility experienced anxiety about the future. They seemed to lose interest in life, their hopes and plans for the future. Many people who go through loss that they experience as devastating end up with negative behaviours such as their use of alcohol (Glatt 2018:105).

Loneliness differed from one person to the next. Lagoy (2021) confirms that loneliness is dependent on what an individual ‘needs and desires’. The quality of family connections, determined by positive factors such as advice and care, affected the holistic well-being of participants. Loss of certainty could affect many aspects of life (McClain 2019).

It was found that self-confidence was needed to give individuals a sense of positivity about their self-worth. The family is the primary means of building the self-esteem of a child and improvement of a child’s capacities. Participants evaluated themselves and mentioned that their self-esteem was affected as individuals in a family. Self-esteem depends on a few perspectives, among which are self-evaluation and the criticism we get from others (Brennan 2020).

In this process of change, grieving people almost always need the support of others. The common thread through the stages of grief was change. Not only did youth had to change inwardly but had to make changes in their daily life and routines, leading to a measure of adjustment and disruption which were hard to deal with.

Limitations of the study were that the study was conducted at only one of the comprehensive primary healthcare facilities in the urban area of Cape Town. As the study was qualitative in nature, the findings cannot be generalised to the broader community of the youth in the urban district. It was difficult to find men to participate in the study. This could be contributed to women who were mainly the bread winners in the family. It is recommended that further research be conducted on the social impact on the youth who are affected by death related to HIV.

## Recommendations

Certain behaviour may help to improve the well-being of individuals who are suffering because of the loss of a family member. Youth should return to the comprehensive primary healthcare clinic to make an appointment with the nurse in order to be referred to a clinical psychologist or pastoral psychologist. The nurse should allow the youth to talk about their fears and cry if necessary, as this is part of the grieving process. Allowing the individuals to express their feelings is very important that can be carried out by asking to join a support group. Youth should provide social services to assist them where they had to take on parental roles, and need assistance with schooling and further studies.

A natural reward approach could include, to understand, that there is not one process and phases going through grief. Youth will at the end of grief, depending of their unique situation, be able to demonstrate that they have changed their guilt and negative feeling (if present). Youth will at a later stage move in a positive direction (e.g. finding a job) and acknowledge their value to support the family. As part of self-management of stress, youth should set up a weekly calendar to ensure that they get enough sleep, exercise, and eat healthy. This will help in their healing and restoration process and will facilitate personal growth, thus promoting not only mere coping and survival but also remarkable strength, growth and resiliency (Abi-Hashem 2017:251).

Assistance to develop a positive mindset is crucial for youth. The youth should be encouraged to share the ways in which they can channel the energy towards, for example, engaging in community activities. Through talking about HIV, it is important that youth realise that HIV is part of life, and that it is not linked to age, social economic status, religion, race or intelligence.

## Conclusion

The death experience, especially of a parent or a close family member, was considered as one of the most traumatic events in a person’s life. Some people experience a sense that the death was not right, and it should not have happened. Common emotions triggered by loss included emotional pain, a feeling of being astounded by the loss, difficulty in accepting the loss and intense longing for the deceased. The deceased was seen as the anchor in the family, and youth should become more aware that it is normal to go through the different stages of the grieving process, and that there is no time frame attached to each stage of grief – it varies from person to person, depending on how close you were with the person who passed away.
